# Clinical efficacy of botulinum toxin type A in patients with traumatic brain injury, spinal cord injury, or multiple sclerosis: An observational longitudinal study

**DOI:** 10.3389/fneur.2023.1133390

**Published:** 2023-04-06

**Authors:** Alessio Baricich, Marco Battaglia, Daria Cuneo, Lucia Cosenza, Marzia Millevolte, Michela Cosma, Mirko Filippetti, Stefania Dalise, Valentina Azzollini, Carmelo Chisari, Stefania Spina, Nicoletta Cinone, Lorenza Scotti, Marco Invernizzi, Stefano Paolucci, Alessandro Picelli, Andrea Santamato

**Affiliations:** ^1^Physical and Rehabilitation Medicine, Department of Health Sciences, Università del Piemonte Orientale, Novara, Italy; ^2^Physical and Rehabilitation Medicine, “Ospedale Maggiore della Carità” University Hospital, Novara, Italy; ^3^Physical and Rehabilitation Medicine, A.S.L. Vercelli, Vercelli, Italy; ^4^Rehabilitation Unit, Department of Rehabilitation, “Santi Antonio e Biagio e Cesare Arrigo” National Hospital, Alessandria, Italy; ^5^Neurorehabilitation Clinic, Department Neurological Sciences, University Hospital of Ancona, Ancona, Italy; ^6^Neuroscience and Rehabilitation Department, Ferrara University Hospital, Ferrara, Italy; ^7^Department of Neurosciences, Biomedicine and Movement Sciences, University of Verona, Verona, Italy; ^8^Neurorehabilitation Section, Department of Translational Research and New Technologies in Medicine and Surgery, University of Pisa, Pisa, Italy; ^9^Spasticity and Movement Disorder Unit, Physical Medicine and Rehabilitation, Policlinico Riuniti, University of Foggia, Foggia, Italy; ^10^Department of Translational Medicine, Unit of Medical Statistics, Università del Piemonte Orientale, Novara, Italy; ^11^Dipartimento Attività Integrate Ricerca e Innovazione (DAIRI), Translational Medicine, “Santi Antonio e Biagio e Cesare Arrigo” National Hospital, Alessandria, Italy; ^12^IRCCS Fondazione Santa Lucia, Rome, Italy

**Keywords:** botulinum toxin, pain, spasticity, movement disorders, multiple sclerosis, spinal cord injury, traumatic brain injury, rehabilitation

## Abstract

Botulinum toxin type A (BoNT-A) is the treatment of choice for focal spasticity, with a concomitant effect on pain reduction and improvement of quality of life (QoL). Current evidence of its efficacy is based mainly on post stroke spasticity. This study aims to clarify the role of BoNT-A in the context of non-stroke spasticity (NSS). We enrolled 86 patients affected by multiple sclerosis, spinal cord injury, and traumatic brain injury with clinical indication to perform BoNT-A treatment. Subjects were evaluated before injection and after 1, 3, and 6 months. At every visit, spasticity severity using the modified Ashworth scale, pain using the numeric rating scale, QoL using the Euro Qol Group EQ-5D-5L, and the perceived treatment effect using the Global Assessment of Efficacy scale were recorded. In our population BoNT-A demonstrated to have a significant effect in improving all the outcome variables, with different effect persistence over time in relation to the diagnosis and the number of treated sites. Our results support BoNT-A as a modifier of the disability condition and suggest its implementation in the treatment of NSS, delivering a possible starting point to generate diagnosis-specific follow-up programs.

**Clinical trial identifier:**

NCT04673240.

## 1. Introduction

The latest definition of spasticity was recently supplied by Li et al. (1) being the increased resistance to externally imposed muscle stretch depending on lengthening velocity and muscle length that results from hyperexcitable descending excitatory brainstem pathways and from the resultant exaggerated stretch reflex responses (1). Although based on post stroke spasticity (PSS), the concept can be extended to other populations affected by upper motor neuron syndrome (UMNS).

Additionally, Pandyan and colleagues focused their definition on the central sensory-integrated control of movement, describing spasticity as “*a disordered sensori-motor control, resulting from an upper motoneuron lesion, presenting as intermittent or sustained involuntary muscle activation*” (2).

Essentially, the concept of spastic paresis forms part of the more comprehensive picture referred to as UMNS, which often includes other positive clinical manifestations such as flexor (or extensor) spasm, clasp knife phenomenon, Babinski sign, exaggerated cutaneous withdrawal reflexes, autonomic hyperflexia, dystonia, and contractures which may limit voluntary movement and cause discomfort. Moreover, several negative features are also included in UMNS, such as paresis, lack of dexterity, and fatigability.

UMNS can be found in several central nervous system disorders such as stroke, cerebral palsy, multiple sclerosis (MS), traumatic brain injury (TBI), spinal cord injury (SCI), anoxic brain damage, amyotrophic lateral sclerosis, and several metabolic disorders. The clinical presentation may vary, depending on the damage localization, affecting predominantly the antigravity muscles and following typical patterns (3, 4).

It has been emphasized that decreased reciprocal Ia inhibition of α motor neurons *via* disynaptic interneuron and decreased non-reciprocal lb inhibition can have a significant role in spasticity development (5). In addition, sprouting and new connections could influence the emergence of increased stretch reflexes (6, 7). Moreover, environmental and spatial factors such as temperature, time of the day, fatigue, and posture can also influence the severity of spasticity (8). However, the neurobiology of spasticity is still not completely clear.

In the context of UMNS, spasticity figures among the most severe causes of disability since it affects all the domains of the International Classification of Functioning, Disability, and Health (ICF) drafted by the WHO (9). In fact, it can lead to joint deformity, pain, and reduced quality of life (QoL), interfering with the rehabilitation program, emphasizing the necessity of correct clinical assessment and therapeutic management.

The clinical approach to spasticity needs to be multimodal (10, 11). The choice of medications directly depends on spasticity distribution: a systemic treatment with oral or intrathecal drugs is generally considered in case of generalized spasticity, whereas local treatments are considered in case of focal spasticity.

In particular, Botulinum Neurotoxin Type A (BoNT-A) is considered the gold standard treatment for focal spasticity, showing level A evidence in both upper and lower limbs (12, 13). BoNT-A provides transient and reversible presynaptic chemodenervation on the neuromuscular plate. Moreover, BoNT-A shows an optimum safety profile and effectiveness in reducing pain, improving walking ability, mobility, nursing procedures, activities of daily living, and ultimately QoL (14), without significantly altering muscle quality (15). However, the current literature is mainly based on post-stroke spasticity (PSS) (16, 17), whereas it is still limited in case of other clinical conditions related to non-stroke spasticity (NSS), such as SCI, TBI, and MS, even though spasticity is a major concern for rehabilitation in these patients.

Considering MS, approximately 80% of patients suffer from spasticity (18): mild, moderate, and severe spasticity were observed respectively in 27.3, 44.0, and 28.7% of MS patients (19). Lower limbs are more severely affected, involving between one half to two thirds of MS patients (20, 21). It should be noted that MS-related spasticity has a fluctuating intensity and can increase during the night (22).

In subjects affected by SCI, during the first 6–12 months after spinal cord lesion, 70% of subjects develop spasticity (23–25), whereas it is present in the chronic phase (>1 year post injury) in 65–78% of patients (24, 26).

In patients with TBI, spasticity onset is typically rapid and can be observed as soon as 1 week after the injury, constituting one of the most relevant barriers to early rehabilitation procedures (27).

Therefore, it is of crucial importance to note that an improvement in spasticity severity, regardless of the etiology, cannot be hypothesized in the absence of a clinical intervention. To this end, a specific treatment must be considered (18, 27, 28).

The primary endpoint of this study is the evaluation of the clinical efficacy of BoNT-A in reducing spasticity, measured with the modified Ashworth scale (MAS), in patients affected by SCI, TBI, and MS, and the duration of the therapeutic effect over time (1 and 3 months after injection).

The secondary endpoints are the evaluation of the treatment-goal achievement by the physician and the patients/caregivers through the Global Assessment of Efficacy scale (GAE), the pain variation from baseline using the Numeric Rating Scale (NRS), and the improvement of QoL using the Euro Qol Group EQ-5D-5L. Finally, the optimal interval between reinjections will be estimated through the monitoring of the trends of the above variables, including a follow-up visit 6 months post injection.

## 2. Material and methods

This is an observational, multicentre, non-interventional, prospective cohort study.

The study population was enrolled among adult patients affected by spastic hypertonia as a consequence of TBI, SCI or MS, already addressed to our centers for the periodical focal spasticity treatment with BoNT-A. The three pharmaceutical forms of BoNT-A approved for clinical use in Italy were included (AbobotulinumtoxinA, OnabotulinumtoxinA, and IncobotulinumtoxinA).

Participation to the study was subordinate to the clinical indication to perform BoNT-A injections.

Due to the absence of other equivalent treatment options for focal spasticity and the already demonstrated high level of BoNT-A efficacy in reducing focal spasticity, a control group was not included, and an observational study design was preferred to ensure an active and approved treatment option. BoNT-A dose was chosen based on spasticity severity, muscle mass, number of treated sites, treatment goals, and specific clinical examination, making it strictly patient-tailored. Furthermore, every BoNT-A product included in this study had equal levels of evidence in treating adult spasticity (Level A for every BoNT-A product considered), making it unnecessary to investigate any relative superiority in the context of this research (12, 13). Therefore, subgroup analysis based on BoNT-A dose or pharmaceutical form was not performed.

Total BoNT-A doses ranged from 500 to 1500 UI for AbobotulinumtoxinA and from 100 to 800 UI for OnabotulinumtoxinA and IncobotulinumtoxinA.

For study participation, all the patients gave their written informed consent structured according to the Declaration of Helsinki. Written informed consent was also obtained from the patients to publish this paper. The study was conducted according to the guidelines of the Declaration of Helsinki and approved by the local Ethics Committee (CE register number 162/18) and the Competent Authority (Ospedale Maggiore della Carità University Hospital, Novara, Italy. Protocol validated on 12 September 2018). This study has been registered on ClinicalTrials with the identifier NCT04673240.

The inclusion criteria were an age >18 years old, diagnosis of MS, SCI, or TBI confirmed clinically and through radiological imaging (magnetic resonance imaging and/or computerized tomography scan), the presence of spasticity graded at least MAS 1+ and requiring medical intervention, and BoNT-A naïvity or at least a 4-month interval between the last injection and the study inclusion.

Exclusion criteria were the presence of fixed contractures or bone deformities in the affected limbs, changes in any oral antispastic medication or in the specific rehabilitation regimen 4 months prior to study entry or during the study, other concomitant neurological or orthopedic conditions involving the affected limbs, and documented sensitivity to BoNT-A or to its excipients.

Muscle tone was assessed with MAS (29, 30). This scale was chosen as the most common assessment of spasticity with an adequate inter- and intra-rater reliability, although it does not consider the elongation velocity. The evaluation of treatment-goal achievement by the physicians, patients, and caregivers after BoNT-A injection was performed with GAE (31). Pain intensity was measured using NRS (32). In cases of multisite injection, the highest NRS value was considered. Quality of life was assessed with the Euro Qol Group version (EQ-5D-5L) (33), both in its descriptive system (EQ 5D), based on five dimensions and five levels, and the EQ visual analog scale (EQ VAS), which records the patient's self-rated health on a scale from 0 to 100. The EQ VAS can be used as a quantitative measure of health outcome that reflect the patient's own judgement.

Post-injection treatment did not differ from normal clinical practice.

Patients were assessed in three sessions: at the baseline before ultrasound-guided BoNT-A injection (T0), after 1 month (T1), and 3 months (T2) from injection.

An additional follow-up visit was performed at 6 months from baseline (T3) only in subjects who, based on the clinical re-evaluation, did not receive reinjection at T2. This adjunctive check was included to monitor the persistence of the therapeutic effects.

The choice of this timeline was based on the pharmacological activity of BoNT-A which has a peak effect at 1 month, a possible initial decline at 3 months, and a maximal therapeutic duration of 6 months (16, 34). Reinjection is usually considered from 3 months, based on the drug data sheets and the Food and Drug Administration's statements. These data are mainly based on PSS literature.

**Figure 1:**
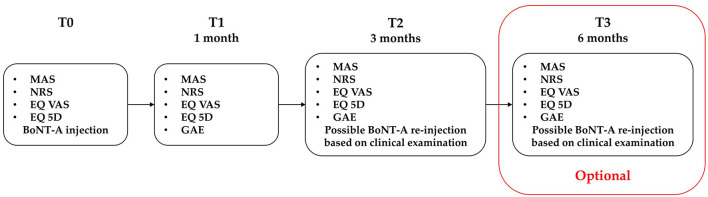
Study flowchart. MAS, modified Ashworth scale; NRS, numeric rating scale; EQ VAS, European quality of life visual analog scale; EQ 5D, European quality of life 5 dimensions. An optional T3 visit was performed for subjects who did not receive second BoNT-A treatment at T2.

The study timeline is described in Figure 1.

Considering the previous results obtained in the literature with BoNT-A treatment, the sample size was calculated focusing on the primary outcome measure (a reduction in spasticity at 1 and 3 months), assuming an expected 75% of prevalence for clinically relevant spasticity in these populations (18, 19, 23–26). Being a prospective study, the following formula for qualitative variables was proposed: [1,96 × (P × (1-P))/0.01 (35). Being *P* = 75% and assuming an absolute error of 0.1 and a type I error of 0.05, the total estimated sample size obtained was 72.

Descriptive statistics were used to summarize patients' characteristics. Categorical variables were reported as absolute frequencies and percentages, and continuous variables as mean and standard deviation (SD). Four mixed effect linear regression models were fitted to evaluate the change over time of MAS, NRS, VAS Qol, and EQ5D scores adjusted for type of diagnosis and number sites treated. A random intercept related to patients was included in the model to account for repeated measures within patients. Subsequently, for each outcome measure, the models were fitted with two additional interaction terms, the first one between time and diagnosis and between time and the number of sites treated, and specific contrasts were performed to evaluate the change of the outcomes' measures compared to T0 stratified by diagnosis and number of sites. The same models were fitted for the subgroup of subjects that did not have repeated injection at T2. Given the large number of tests performed, the false discovery rate adjustment was applied to account for the potential inflation of type I errors. For VAS QoL and EQ5D scores, the minimally clinically important difference (MCID) at T1 was calculated as 1.96^*^√2^*^SEM, where SEM is the standard error of measurements. Finally, the concordance between the GAE scores reported by the patients and operator at T1 was evaluated using the Cohen's kappa statistic. All tests were two-tailed and the type I error was set to 0.05. Statistical analyses were performed using SAS version 9.4 (SAS Institute, Cary NC).

## 3. Results

In total, 100 patients were pre-screened to participate to the study, and 86 met the inclusion criteria and received BoNT-A injection at T0 with the panel of basal evaluations. Two and five patients dropped out at T1 and T2, respectively. A total of 79 subjects completed the study, meeting the minimum sample size required. No adverse effects were recorded after injection. A total of 48 patients did not receive a second BoNT-A treatment at T2 based on the clinical re-evaluation. For this subgroup, an adjunctive visit (T3) at 6 months from the first injection was considered. Demographic and interventional characteristics are described in Table 1.

**Table 1:** Demographic characteristics of the study population.

	***N =* 86**
	***N* (%)**
**Sex**	
M	49 (57)
F	37 (43)
Age, mean (DS)	47.45 (12.56)
**Diagnosis**	
MS	48 (55.81)
SCI	18 (20.93)
TBI	20 (23.26)
**BoNT-A product**	
OnabotulinumtoxinA	16 (18.60)
AbobotulinumtoxinA	52 (60.47)
IncobotulinumtoxinA	18 (20.93)
**Body site**	
Hip adductors Dx	27 (31.34)
Hip adductors Sx	24 (27.91)
Hip flexors Dx	5 (5.81)
Hip flexors Sx	6 (6.98)
Plantar flexors Dx	39 (45.35)
Plantar flexors Sx	23 (26.67)
Knee flexors Dx	22 (25.58)
Knee flexors Sx	18 (20.93)
Elbow flexors Dx	12 (13.95)
Elbow flexors Sx	6 (6.98)
Wrist flexors Dx	7 (8.14)
Wrist flexors Sx	5 (5.81)
Shoulder adductors Dx	8 (9.30)
Shoulder adductors Sx	8 (9.30)
**N**°**of treated sites**	
1	25 (29.07)
2	24 (27.91)
≥3	37 (43.02)

MS, multiple sclerosis; SCI, spinal cord injury; TBI, traumatic brain injury. Data are referred to T0.

Our results showed a significant effect of BoNT-A in reducing spasticity and pain persisting up to T2 in the general population. Similarly, a significant improvement of the EQ VAS and the EQ 5D could be seen up to T2. The results can be seen in Table 2.

**Table 2:** Beta regression model of time effect on the considered variables (MAS, NRS, EQ VAS, and EQ-5D) up to T2.

	**MAS**		***F*-test**	**NRS**		***F*-test**
**Time**	**beta (se)**	***p*-value**	***p*-value**	**beta (se)**	***p*-value**	***p*-value**
**T0**	ref			ref		
**T1**	−0.99 (0.10)	**< 0.0001**	**< 0.0001**	−1.69 (0.25)	**< 0.0001**	**< 0.0001**
**T2**	−0.51 (0.10)	**< 0.0001**		−1.03 (0.26)	**< 0.0001**	
	**EQ VAS**		***F*** **-test**	**EQ 5D**		***F*** **-test**
**Time**	**beta (se)**	***p*** **-value**	***p*** **-value**	**beta (se)**	**beta (se)**	***p*** **-value**
**T0**	ref			ref		
**T1**	7.56 (1.7)	**< 0.0001**	**< 0.0001**	0.06 (0.02)	**0.0004**	**0.0016**
**T2**	4.63 (1.73)	**0.0083**		0.03 (0.02)	**0.0441**	

The Fisher test was used to assess the relationships between the outcome variables. MAS, modified Ashworth scale; NRS, numeric rating scale; EQ VAS, European quality of life visual analog scale; EQ 5D, European quality of life 5 dimensions; beta: mean value of variation of the dependent variable in relation to independent variable modification; F**-**test, Fisher test; se, standard error. Significant variations are in bold. Significance level p < 0.05.

Subgroup analysis confirmed the efficacy of BoNT-A in reducing MAS at T1 in all the diagnosis groups, with an effect persistence at T2 for MS and TBI patients. Pain reduction accurately mimicked the MAS trend, while QoL was significantly improved only in MS patients and only in the EQ VAS evaluation. Interestingly, patients' stratification per number of treated sites showed a higher QoL improvement both in the EQ VAS (at T1 and T2) and EQ 5D (at T1) in subjects treated in three or more sites. The results are described in Table 3.

**Table 3:** Modification of variables' mean values assessed with the false discovery rate (FDR) method, stratified per diagnosis and number of treated muscles.

	**MAS**				**NRS**			
**Diagnosis**	**Δ T1-T0**	***p*-value**	**Δ T2-T0**	***p*-value**	**Δ T1-T0**	***p*-value**	**Δ T2-T0**	***p*-value**
MS	−0.88 (0.13)	**< 0.0001**	−0.50 (0.13)	**0.0004**	−1.73 (0.33)	**< 0.0001**	−1.11 (0.34)	**0.0025**
SCI	−0.78 (0.21)	**0.0004**	−0.40 (0.21)	0.0585	−1.28 (0.54)	**0.0219**	−0.50 (0.56)	0.3728
TBI	−1.44 (0.19)	**< 0.0001**	−0.64 (0.20)	**0.0020**	−1.95 (0.51)	**0.0005**	−1.29 (0.53)	**0.0219**
	**EQ VAS**				**EQ 5D**			
**Diagnosis**	Δ **T1-T0**	***p*** **-value**	Δ **T2-T0**	***p*** **-value**	Δ **T1-T0**	***p*** **-value**	Δ **T2-T0**	***p*** **-value**
MS	8.33 (2.28)	**0.0021**	5.48 (2.30)	0.0546	0.05 (0.02)	0.078	0.04 (0.02)	0.0834
SCI	6.22 (3.64)	0.1224	0.85 (3.79)	0.8226	0.08 (0.04)	0.078	−0.02 (0.04)	0.6505
TBI	6.94 (3.52)	0.1013	5.93 (3.61)	0.1224	0.07 (0.03)	0.078	0.07 (0.04)	0.0834
**N**°**sites**								
1	4.62 (3.11)	0.2094	1.36 (3.28)	0.6784	0.07 (0.03)	0.0833	0.07 (0.03)	0.0843
2	5.89 (3.17)	0.1304	2.41 (3.17)	0.5377	0.04 (0.03)	0.3013	0.01 (0.03)	0.7175
≥3	10.60 (2.57)	**0.0004**	8.17 (2.59)	**0.0059**	0.07 (0.03)	**0.0413**	0.03 (0.03)	0.3052

MS, multiple sclerosis; SCI, spinal cord injury; TBI, traumatic brain injury; EQ VAS, European quality of life visual analog scale; EQ 5D, European quality of life 5 dimensions. Significant variations are in bold. Significance level p < 0.05.

Considering the 48 subjects (MS: *n* = 29, 60%; SCI: *n* = 11, 23%; TBI: *n* = 8, 17%) who did not receive a second BoNT-A treatment at T2, the results showed a persistence of the therapeutic effect in reducing spasticity lasting up to T3, with a return to basal conditions for the NRS, the EQ VAS, and the EQ 5D. The results are shown in Table 4.

**Table 4:** Beta regression model of time effect on the considered variables (MAS, NRS, EQ VAS, and EQ 5D) up to T3.

	**MAS**		**F-test**	**NRS**		**F-test**
**Time**	**beta (se)**	***p*-value**	***p*-value**	**beta (se)**	***p*-value**	***p*-value**
**T0**	ref			ref		
**T1**	−0.95 (0.12)	**< 0.0001**	**< 0.0001**	−1.93 (0.35)	**< .0001**	**< 0.0001**
**T2**	−0.76 (0.12)	**< 0.0001**		−1.46 (0.35)	**< .0001**	
**T3**	−0.29 (0.12)	**0.0171**		−0.37 (0.36)	0.3031	
	**EQ VAS**		**F-test**	**EQ 5D**		**F-test**
**Time**	**beta (se)**	***p*** **-value**	***p*** **-value**	**beta (se)**	***p*** **-value**	***p*** **-value**
**T0**	ref			ref		
**T1**	6.45 (1.72)	**0.0003**	**0.0005**	0.05 (0.02)	**0.0038**	**0.0292**
**T2**	6.31 (1.72)	**0.0003**		0.03 (0.02)	0.0574	
**T3**	3.1 (1.76)	0.0798		0.02 (0.02)	0.2579	

The Fisher test was used to assess the relationships between the outcome variables. MAS, modified Ashworth scale; NRS, numeric rating scale; EQ VAS, European quality of life visual analog scale; EQ 5D, European quality of life 5 dimensions; beta: mean value of variation of the dependent variable in relation to independent variable modification; F test: Fisher test; se, standard error. Significant variations are in bold. Significance level p < 0.05.

The evaluation of treatment goal achievement with GAE after BoNT-A injection showed an overall satisfactory perceived result reported by both the patients/caregivers and the physicians. At T1, a positive (very good or good) response rate of 73.81 and 83.25% was recorded among patients/caregivers and physicians, respectively, and at T2 the positive response rates were 70.51 and 75.64%, respectively, as seen in Table 5. Moreover, a good correlation between the two measurements was found at T1 [Kappa weighted index: 0.64 (0.51–0.77)] and a moderate correlation at T2 [Kappa weighted index: 0.47 (0.32–0.63)]. The correlation significance is shown in Table 6.

**Table 5:** BoNT-A treatment efficacy as reported by the patients/caregivers and the physicians.

		**Patient/caregiver**		**Physician**	
		**T1**	**T2**	**T1**	**T2**
**GAE**		***N* (%)**	***N* (%)**	***N* (%)**	***N* (%)**
**1**	Very good	22 (26.19)	14 (17.95)	17 (20.24)	8 (10.26)
**2**	Good	40 (47.62)	41 (52.56)	53 (63.1)	51 (65.38)
**3**	Moderate	20 (23.81)	19 (24.36)	13 (15.48)	18 (23.08)
**4**	Poor	2 (2.38)	4 (5.13)	1 (1.19)	1 (1.28)
**Missing**		2	8	2	8

GAE, global assessment of efficacy scale.

**Table 6:** Correlation between physician- and patient/caregiver-reported efficacy after BoNT-A treatment at T1 and T2.

**T1**	**Physician GAE**					**T2**	**Physician GAE**				
**Patient/ caregiver GAE**	**1**	**2**	**3**	**4**	**Total**	**Patient/ caregiver GAE**	**1**	**2**	**3**	**4**	**Total**
**1**	15	7	0	0	22	**1**	6	8	0	0	14
**2**	2	37	1	0	40	**2**	2	34	5	0	41
**3**	0	8	11	1	20	**3**	0	7	11	1	19
**4**	0	1	1	0	2	**4**	0	2	2	0	4
**Total**	17	53	13	1	84	**Total**	8	51	18	1	78

Kappa weighted index was used to assess correlation significance. At T2, missing data lead to a total of 78 patients. GAE, global assessment of efficacy scale.

## 4. Discussion

In the overall population, the results showed a significant clinical effect of BoNT-A in reducing spasticity for up to 3 months after injection. Similarly, pain and QoL measured with both the EQ 5D and EQ VAS appeared to be improved from T0 until T2.

The main guidelines currently available suggest performing a clinical re-evaluation of patients 3 to 6 months after injection, allowing a potential reinjection after a minimum of 3 months (16). This indication, although based on PSS, could be taken into consideration even in the context of NSS as a starting point to create more focused follow-up programs. Our results provide reliability to this assumption regarding the effect duration of BoNT-A even in the context of NSS.

Interestingly, subgroup analysis gave an additional and more specific evaluation of how patients affected by different UMNS-related clinical conditions responded to BoNT-A treatment. In particular, the reduction of MAS was significant at T1 in every sub-population and at T2 for MS and TBI patients. In SCI subjects, a return to basal conditions could be seen at T2.

This difference in the duration of the pharmacological effect may be due to several features of spasticity in the context of SCI. Firstly, after a spinal cord injury spasticity in most cases is generalized, thus requiring a multimodal approach which may include other pharmacological interventions in association with BoNT-A. Secondly, the presence of “extrinsic spasticity”, common in SCI alongside intrinsic mechanisms, is characterized by complex muscle group activation in response to sensory or noxious stimuli afferent to joints, skin, or muscles themselves. The activation of polysynaptic spinal reflexes can lead to a withdrawal reflex hardly controlled by focal treatment with BoNT-A alone (36). Palazón-García et al. (37) supported our findings regarding the efficacy of BoNT-A in SCI spasticity, emphasizing the need for its correct integration with systemic antispastic drugs and a patient-tailored panel of non-pharmacological interventions.

In the case of MS patients, our findings showed the efficacy of BoNT-A in reducing spasticity, as described by Safarpour and colleagues, confirming the level of evidence, in particular of AbobotulinumtoxinA, regarding improving hygiene and mobility (22). However, the efficacy of BoNT-A in treating spasticity from different causes is not fully cleared. In research based on 99 participants affected by MS, stroke, or cerebral palsy, a higher dose of BoNT-A was necessary to reduce spasticity in MS patients (38). Differently, a study carried out on a larger sample size detected no differences in the outcome measurements in adult spasticity with different etiologies (39). Interestingly, both studies used only OnabotulinumtoxinA.

Regarding TBI spasticity, an early onset can be recorded typically from 3 to 6 weeks after lesion, but it is common to see even earlier cases after just 1 week. Notably, distribution patterns may vary significantly from unilateral to bilateral and from focal to generalized (27). Among the causes of NSS we considered, TBI is the most comparable to PSS and, as a matter of fact, BoNT-A has been demonstrated to have a similar efficacy among these populations. In this regard, Gracies and colleagues showed how AbobotulinumtoxinA can significantly reduce MAS in a group of post stroke and TBI patients compared to placebos, improving functional and global assessment scores also (40). However, the data specifically about BoNT-A efficacy on TBI spasticity are scant and rely on small samples.

Given the equally high level of evidence in reducing adult focal spasticity of the three BoNT-A products we considered (12) and their well-established efficacy and safety profiles (13), the authors considered further patient stratification by BoNT-A pharmaceutical form and the vs. placebo analysis unnecessary.

Considering the other outcome variables, the NRS followed the MAS pattern with a statistically significant correlation, tracing both in the general population and in the subgroup analysis the exact same significancy trend at every time point. This outcome supports the current evidence of the antalgic effect of BoNT-A and the pain relief consequent to the pharmacological effect and spasticity reduction (41, 42). These assumptions, based on general UMNS literature, appear to be worthy even for our population, confirming the role of BoNT-A in modulating both nociceptive/mechanical and neuropathic pain even though the precise antalgic effect needs to be clarified. In particular, the neuromodulating role of BoNT-A, carried out through the inhibition of neurotransmitters release, such as substance P, glutamate, and calcitonin gene-related peptide, was recently re-confirmed in the treatment of chronic migraines and other forms of primary headaches (43). Even in the context of spasticity management, BoNT-A showed an interesting effectiveness in reducing hemiplegic shoulder pain. Tan B. and Jia L. suggested that the correct targeting of the subscapularis muscle with an ultrasonographic guide and an adequate BoNT-A dose and dilution could provide a significant reduction in spasticity and pain with an improvement of QoL (44), supporting the mechanical component of spasticity-related pain. However, the current scientific literature provides limited evidence about a defined antalgic role of BoNT-A in the population we considered, and guidelines on neuropathic pain management are yet to be drafted even though the existing findings are promising (41, 45). Furthermore, pain has a relevant psychosocial component, and the presence of a placebo effect should be considered with specific trials. To this end, the presented results may be considered a starting point to a deeper understanding of the complex interdependence of pain and spasticity in MS, SCI, and TBI patients.

Additionally, the reduction of spasticity and pain could significantly improve the range of motion, motility, walking capability, upper and lower limb functional integration, and, in totally dependent patients, aid easier and optimized caregiving (13). From the point of view of Physical and Rehabilitation Medicine specialists, the temporary reduction of spasticity and pain provided by BoNT-A can allow an “operating window” to open in which the rehabilitative treatment could be more impactful and effective, optimizing the residual functional resources of patients. To this end, it is fundamental to consider BoNT-A treatment as a part of a multimodal therapeutic approach to patients.

In this context, the present study aimed to provide indications for appropriate re-evaluation and re-injection timings in order to avoid an oscillating pattern of spasticity and of the subsequent disability conditions, providing instead a more stable functional status over time.

Regarding the QoL, the current scientific literature supports in most cases the role of BoNT-A in the reduction of disability status (13, 40, 46). Other authors, however, have found no adequate evidence to refute or support the improvement of walking capability and QoL after BoNT-A treatment (47, 48).

Our results showed a significant improvement of QoL in the general study population both in the EQ VAS and in the EQ 5D at T1 and T2. However, subgroup analysis showed fewer promising results; in fact, a relevant increase of QoL could only be seen for MS patients at T1 and in the EQ VAS evaluation. Notably, in patients with a higher number of treatment sites (≥ 3), the improvement of EQ VAS was significant up to T2 and for the EQ 5D up to T1, regardless of the diagnosis. The clinical need for multisite treatment plausibly implies a more severe functional status; therefore, these data displayed a greater modifiability of QoL-related variables in patients with a worse disability condition, suggesting a more impactful role for BoNT-A if implemented in a context of multifocal therapy in more compromised patients.

An interesting observation concerns the evaluation of the treatment goals achievement. This item is, by definition, set regarding the specific clinical condition of patients and tailored on individual needs and improvement expectations. In support of this, recent literature about MS remarked upon the importance of treatment goal setting prior to BoNT-A injection (49), requiring periodical reconsideration at every cycle. Therefore, therapeutic goals are significantly variable among subjects and, especially in case of progressive diseases such as MS, they may significantly vary through time. Both physicians and patients/caregivers were given the same rating scale and, remarkably, in the great majority of cases, reported a positive level of satisfaction (answering “very good” or “good”) after BoNT-A treatment at T1 and T2 with a significant level of correlation between the two observers. Even though GAE might favor positive responses, having three positive and one negative outputs, this information gains significance by being consistent with the EQ 5D and EQ VAS. Therefore, it could provide further internal coherence to our results, supporting the role of BoNT-A in improving the perceived treatment effectiveness alongside the above mentioned and more quantitative measurements of QoL. Additionally, the consistency between the two independently reported scores endorsed the correct achievement of treatment goals.

Finally, an extra follow-up visit after 6 months from injection was considered for a partial number of the population. At T2, 31 patients received additional treatment with BoNT-A based on clinical re-evaluation. The 3 months period from T0 is considered the minimal time lapse between two subsequent cycles according to current literature (16, 17, 34, 50). The remaining 48 subjects did not meet the clinical criteria for re-injection; therefore, it was possible to assess the continuation of BoNT-A effect after 6 months. This population registered a similar diagnostic distribution to the main population, respectively: MS 60%, SCI 23%, and TBI 17%. The results showed a persistence of MAS reduction and a return to the basal conditions of NRS and QoL measurements. Regarding pain, our findings matched the current evidence. The antalgic effect of BoNT-A on neuropathic post stroke and post-SCI pain was demonstrated to last more than 3 months with an undetermined maximal duration (41). In case of mechanical spasticity-related pain, the antinociceptive effect is not expected to last beyond 3 months (44). The QoL aspect is far more elaborate. We considered populations affected by complex forms of disability, influenced by several intrinsic and extrinsic factors and, in the case of MS, the evolutionary nature of the disease must be taken into account. Specifically, regarding MS, additional reassessments should be provided in case of disease evolution and disease modifying therapy adjustments, aiming to maintain regular adherence to BoNT-A treatment (51). Therefore, it is too simplistic to consider just treatment with BoNT-A as a modifier of a patient's disability condition, and an integrated approach should be preferred.

For these reasons, the authors propose performing reinjection after 3 to 6 months from the previous injection, based on a clinical re-evaluation, in order to maintain a stable control of spasticity and pain and preventing unhealthy oscillations of disability status. In the case of SCI patients, an early control visit after 1 month should be proposed. Our results may help to schedule follow-up visits timed also according to the primary diagnosis, which is proven to influence BoNT-A response duration.

In this T3 population, due to a too low sample size, a diagnosis-based subgroup analysis was not performed.

The population considered represents a “real life” spectrum of different disability conditions, with great variation of severity, expected functional outcomes, and treatment rationale. This implies an equally varied range of therapeutic indications, spasticity patterns, and treatment goals. On this basis, and given the clinical aim of this work, the authors considered to overlook the subgroup analysis based on BoNT-A dose stratification that may fail to describe the real disability status and rehabilitation needs.

The authors are aware of the limitations of this study.

Firstly, the sample size, although satisfying the minimum, was relatively small, especially in the context of subgroup analysis.

Secondly, the population was quite heterogenous, and the treatment varied among patients involving both the upper and the lower limbs. However, our analysis concerned mainly patient-centered goals that only partially depended on injection pattern.

Finally, diagnosis-specific scales could have been added. For instance, the American Spinal Cord Injury Association Impairment Scale (AIS) for SCI, the Expanded Disability Status Scale (EDSS) for MS, and the Glasgow Outcome Scale (GOS-E) for TBI could have been implemented. However, the small size of diagnosis subgroups could not have been further stratified.

In conclusion, our findings support the impactful role of BoNT-A in reducing spasticity and pain and in improving QoL with a high level of perceived efficacy and post-treatment satisfaction in our population. However, the effect duration may vary from 3 to 6 months depending on the diagnosis (MS, SCI, or TBI) and, regarding QoL, on the number of treated sites. Therefore, it is fundamental to establish a solid follow-up program with the aim of the stable preservation of function and improved disability care.

The data obtained could be helpful in identifying the tailored timings of clinical re-evaluation and possible reinjection, providing additional evidence about the effects of BoNT-A in the context of NSS.

Future studies may focus on larger samples and take into consideration a possibly different BoNT-A response in each muscle group. Further research is needed in order to better clarify these aspects.

## Data availability statement

The raw data supporting the conclusions of this article will be made available by the authors, without undue reservation.

## Ethics statement

The studies involving human participants were reviewed and approved by the Comitato Etico Interaziendale Novara, corso Mazzini 18, 28100 Novara, Italy. The patients/participants provided their written informed consent to participate in this study.

## Author contributions

AB and AS conceptualized this study. AB curated the methodological implant and administrated the study project. AP and CC validated the study protocol. LS performed the formal analysis. MB, DC, LC, MM, MC, MF, SD, VA, SS, and NC carried out the investigation and provided the study resources. MB, DC, LC, and LS curated data management. MB and DC wrote and prepared the original draft. AB, MB, and MI wrote, reviewed, and edited the final manuscript. AB and MB visualized the final work. AS, AP, MI, SP, and CC supervised the study conduction. AB and SP undertook funding acquisition. All authors contributed to the article and approved the submitted version.
